# Identification of a reverse crossover point during moderate-intensity exercise (>6 h; 69% VO_2max_) in a world-class triathlete—A secondary analysis

**DOI:** 10.3389/fnut.2025.1627404

**Published:** 2025-10-07

**Authors:** Timothy Noakes, Alex Buga, Jeff Volek, Philip Prins

**Affiliations:** ^1^Department of Human Biology, Division of Exercise Science and Sports Medicine, University of Cape Town, Cape Town, South Africa; ^2^Department of Human Sciences, The Ohio State University, Columbus, OH, United States; ^3^Department of Exercise Science, Grove City College, Grove City, PA, United States

**Keywords:** reverse crossover point, triathlon, carbohydrate oxidation, fat oxidation, glycogen

## Abstract

**Figure:**
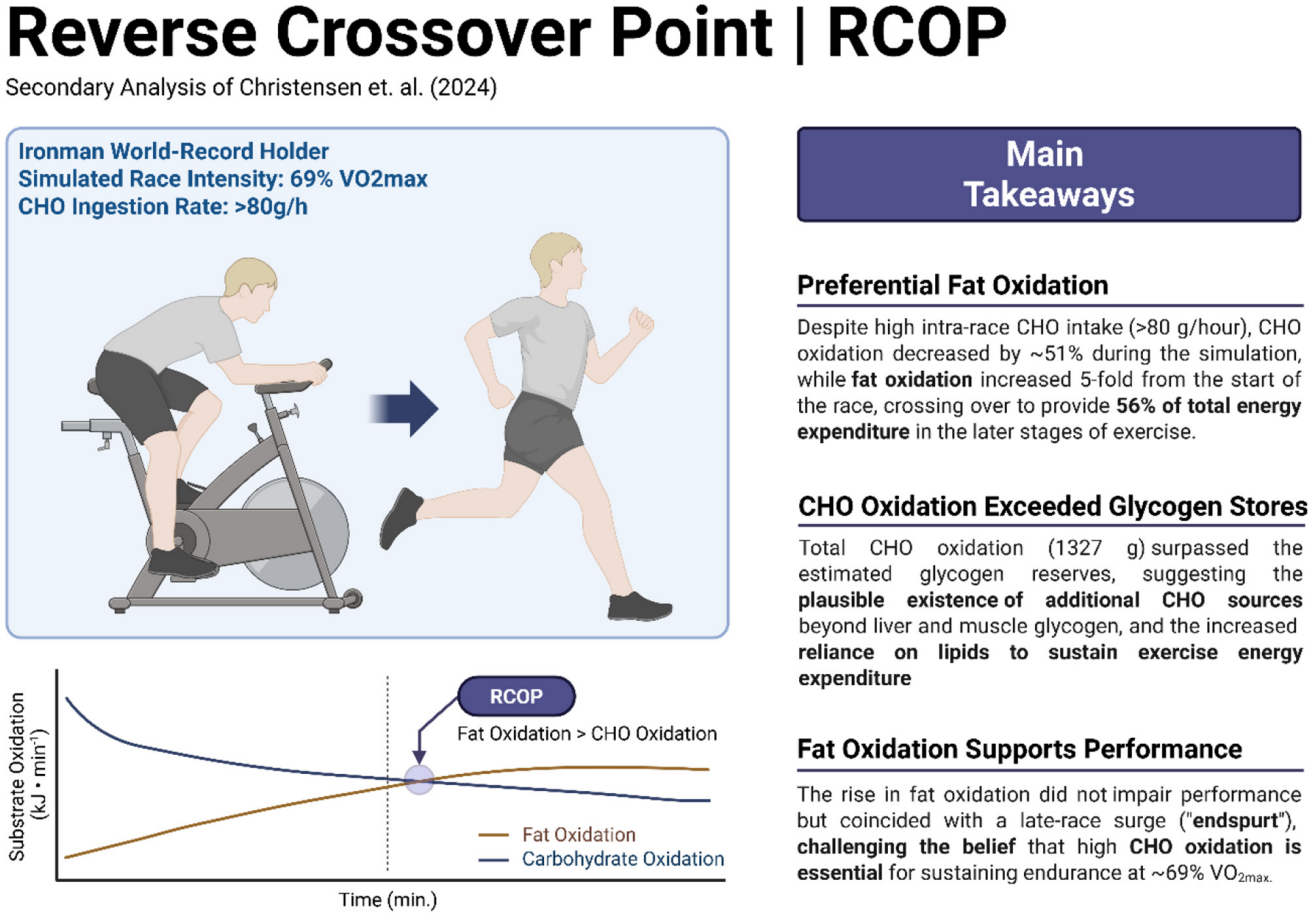
Re-analysis results (Left) and takeaway messages (Right) are summarized above. RCOP, reverse crossover point. Figure created with Biorender.com.

Re-analysis results (Left) and takeaway messages (Right) are summarized above. RCOP, reverse crossover point. Figure created with Biorender.com.

## Introduction

The crossover point (COP) refers to the increase in CHO oxidation with increasing exercise intensity (1) which posits that, at any exercise intensity above 85%, zero energy can come from fat oxidation. This is the basis for the belief that CHO is the obligatory fuel for high intensity exercise (2) and that muscle glycogen depletion in the active muscles is the favored explanation for the fatigue that develops during prolonged exercise (3–5).

We define the reverse crossover point (RCOP) as the point during prolonged, steady-state endurance exercise at which energy from fat oxidation exceeds that from carbohydrate (CHO) oxidation despite maintained exercise intensity. Unlike the conventional COP which reflects a substrate shift due to rising exercise intensity and catecholamine stimulation, the RCOP reflects a time-dependent shift in substrate use, potentially driven by declining glycogen availability, falling insulin levels, and progressive upregulation of lipolysis and fat oxidation pathways (6).

The first study showing this effect was performed in 1923 by H.T. Edwards, R. Margaria and D.B. Dill (7) at the Harvard (University) Fatigue Laboratory. They reported the results from their “‘best subject' runner Y” aged 19, a skilled runner who had finished “near the front in marathons” when he ran for up to 6 h on a laboratory treadmill at speeds of between 9.3 and 11.3 km/h on three separate occasions.

They revealed that Y's blood “sugar” (glucose) concentration was elevated during the first hour of exercise but stayed within the homeostatically-regulated normal range from hours 2 to 5 before falling to a value consistent with hypoglycemia (< 70 mg/100 ml; 3.9 mmol/L) after five and a half hours. The subject's RQ also fell steeply, indicating a progressive shift from CHO to fat metabolism. The result was that Y showed clear evidence for the RCOP already after 180 min when fat oxidation increased to provide 60% of energy [Edwards et al. (7); fig. 3]. After 6 h this increased to 77%.

The authors concluded by describing the RCOP: “as CHO reserve diminishes the proportion of energy derived from fat may increase from 8 to 77 percent.” They also concluded that: “There is no evidence that CHO is more essential in work than in rest”

[Edwards et al. (7), p. 209] and that: “Fat continues to be burned even with a plethora of carbohydrate. From the evidence we cannot conclude that carbohydrate is essential for strenuous exercise” [Edwards et al. (7), p. 208]. The result was that in 6 h Y changed from a high-insulin, glycogenolytic, low-lipolytic to a low-insulin, lipolytic, ketogenic, gluconeogenic (glucose-producing) metabolic state, as happens when one converts from the HCLF to the LCHF diet.

In 1989 Stein et al. (8) reported the presence of the RCOP in four male and four female Ironman triathletes during 3-h of bicycling followed by 5-h of running in a laboratory environment during which their metabolic response was measured also with radio-labeled tracers. The overall exercise intensity maintained during exercise was 53% VO_2max_. Whereas, fat oxidation provided just 30% of the energy expenditure during the first hour of exercise, by the end of the second hour this had already risen to 50%; increasing progressively reaching 77% at the end of the seventh and eighth hours [Stein et al. (8), table 2, 1989, fig. 1] when the athletes mean rate of fat oxidation was 0.95 g/min. Subjects ingested 200 g (25 g/h) CHO as a 5% CHO drink during exercise. The habitual diet of these triathletes was not described.

**Figure 1:**
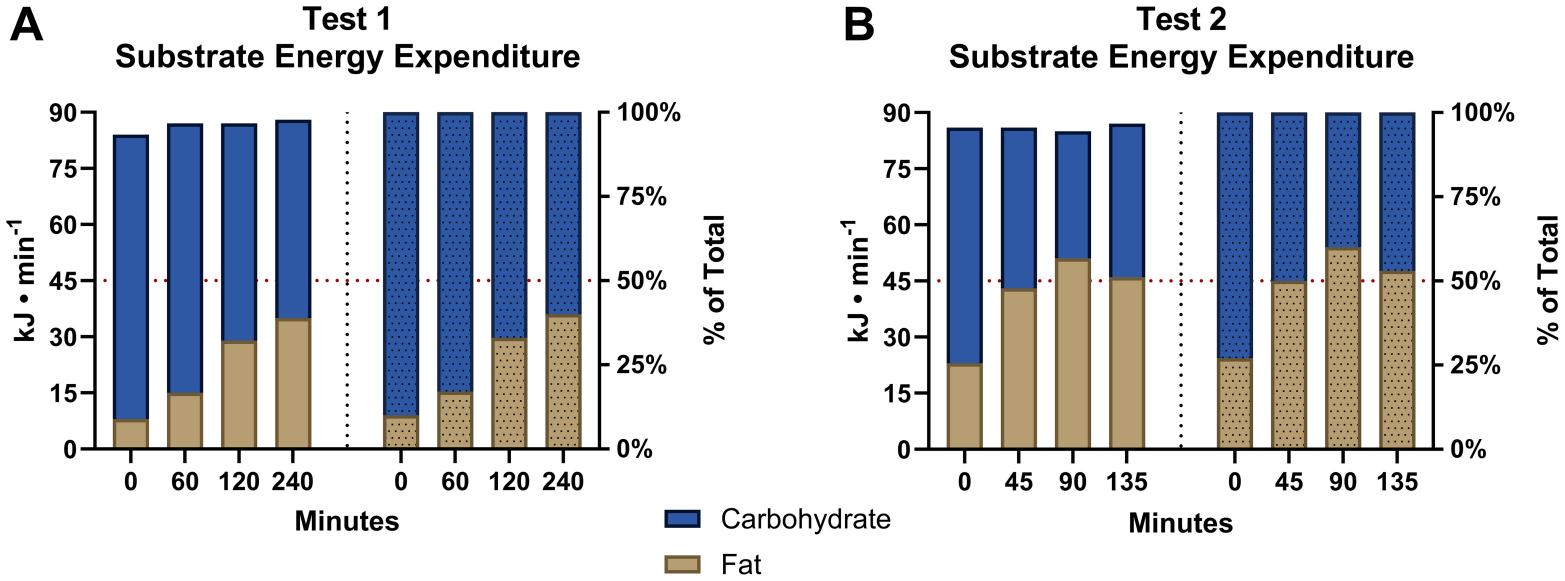
Substrate energy expenditure. Rates of fat and CHO oxidation at specified intervals during the cycling **(A)** and running **(B)** legs of the simulated Ironman Triathlon.

For the past five decades, exercise physiology research has largely examined healthy participants habitually consuming high-CHO diets and intra-exercise CHO solutions. Such individuals typically present with elevated insulinemia and gluconeogenesis, alongside suppressed lipolysis and reduced ketogenesis. Nonetheless, even within this carbohydrate-rich metabolic context, prolonged moderate-intensity, steady-state exercise (>2 h) can elicit the RCOP phenomenon [Watt et al. (9), fig. 1], thereby warranting athletic performance consideration alongside the traditional COP paradigm.

Modern day performances of the top finishers in the 226 km Ironman Triathlon present a significant challenge to the theory that CHO is the obligatory fuel for such prolonged exercise since human athletes may be unable to store sufficient muscle glycogen to provide this obligatory fuel to sustain exercise at this intensity for 7–8 h. Nor may rates of exogenous CHO oxidation be sufficient to maintain appropriately high rates of CHO oxidation once muscle glycogen stores are depleted (10). Thus, it is challenging to reconcile performance in, particularly, the final 42 km run of the 226 km Ironman triathlon with the current explanation which holds that once significant muscle glycogen depletion has occurred, it is not possible to continue performing at moderately high intensity (11). Laboratory data indicate that cyclists riding at 40 km/h should experience near-total muscle glycogen depletion after covering 180 km in 4.5 h (12). Yet in the Ironman Triathlon these athletes must still complete a 42.2 km marathon run.

In 2000, Noakes suggested an alternate solution (11). He proposed that a fat oxidation rate of 1.15 g/min during the marathon running section of the Ironman Triathlon would be sufficient to sustain a running speed of 16 km/h [figures 9 and 10 in Noakes (11)] in athletes who began the final marathon run with glycogen-depleted leg muscles. However, top Ironman Triathlon finishers can sustain a running pace close to 16 km/hr for an additional 160 min when completing the marathon, at an exercise intensity of ~66% VO_2_ max. Studies by Rauch et al. (13) and O'Brien et al. (14) indicate that CHO oxidation during ultra-endurance efforts exceeding 6 h likely surpasses the estimated CHO stores in the active muscles and liver by up to 100%. This discrepancy suggests either inaccuracies in these calculations or the involvement of alternative CHO sources other than in the liver and active muscles to fuel such prolonged exercise.

Volek et al. (15) reported the first major study measuring energy metabolism during 3-h of exercise at 64% VO_2_max in groups of elite ultramarathon runners who had self-selected to eat either the LCHF or the HCLF diet for ~20 months prior to enrolling in the study. Rates of fat oxidation in the LCHF-habituated ultramarathoners were already 1.2 g/min (providing 46 kJ/min) at the start of exercise and remained relatively unchanged for the remainder of exercise. Rates of CHO oxidation also remained essentially unchanged at about 0.4 g/min (7 kJ/min) for the duration of exercise. In contrast, rates of fat oxidation in athletes habituated to the HCLF diet began at 0.48 g/min at the start of exercise, reaching peak fat oxidation rates of 0.82 g/min (32 kJ/min) only after 3 h of exercise during which rates of CHO oxidation, after starting at 1.75 g/min (30 kJ/min) fell to 1.15 g/min (20 kJ/min) at 180 min. This reflected dietary differences in CHO (486 vs. 82 g) and in fat intakes (91 vs. 226 g). In this cohort, the LCHF group appeared to be in approximate CHO balance, as their reported intake during exercise closely matched the estimated rate of CHO oxidation over the 3-h bout. Thus, fat oxidation provided >84% of the energy used by the LCHF-adapted ultramarathoner runners who self-select to eat the LCHF diet. Whereas, in the HCLF-adapted fat oxidation provided just 38% of total energy at the start of exercise, oxidation increased to 61% after 120 min of exercise.

The opinion presented herein is based from an article published by Christensen (16) evidencing a case study simulating a world record performance by the current Ironman Triathlon world record holder (MD) in a near full-distance Ironman Triathlon event. He reported regular measurements of VO_2_ and respiratory quotient (RQ) during 240 min of cycling and 135 min of running, both at world-record Ironman Triathlon pace. The experiment was conducted over 2 days. On the first day the 82 kg subject consumed >10 g/kg body weight CHO (820 g; 13,776 kJ) and completed 46 min of open water swimming at an average heart rate of 161 beats/min. He then cycled for a total of 4 h before running 8.6 km. Overnight, prior to the second day of testing, he ingested a “reduced” CHO diet (i.e., no carbohydrate loading), in contrast to >10 g/kg body weight CHO the day before Test Day 1. The exact CHO intake prior to Day 2 was not quantified in the original study, which limits direct interpretation of pre-exercise glycogen status. He began the day by cycling for 2 h on a stationary bicycle. Thereafter, he began a ~135-min run covering 35 km at his expected race pace. The RCOP phenomenon that occurred during the running portion must be viewed with the understanding that the triathlete followed a habitual HCLF diet, specifying CHO as the dominant macronutrient.

## Methods

The VO_2_ and RQ values were extracted from figure 3 [Christensen (16), fig. 3] by digitizing the plotted data using WebPlotDigitizer (v4.6). Substrate oxidation rates were then calculated using standard equations (17). Specifically (i) rate of total energy expenditure in kilojoules per minute (kJ/min) equals VO_2_ (L/min) × 21; (ii) rates of CHO oxidation (kJ/min) = total energy expenditure (kJ/min) × [(RQ – 0.7)/0.3)]; (iii) rates of CHO oxidation (g/min) = rates of CHO oxidation (kJ/min) divided by 17 (kJ/g CHO); (iv) rates of fat oxidation (kJ/min) are calculated as the total energy expenditure (kJ/min) – energy derived from CHO oxidation (kJ/min); (v) rates of fat oxidation (g/min) are calculated as rates of fat oxidation (kJ/min) divided by 38 (kJ/g fat). The raw data is available in the Supplementary material S1.

## Results

Figure 1 shows rates of CHO and fat oxidation in kJ/min and % of total when measured at the specified intervals during the simulated cycling (Figure 1A) and running (Figure 1B) legs of the simulated Ironman Triathlon. There was a progressive reduction in rates of CHO oxidation beginning from the start of exercise so that at the finish after 375 min of exercise CHO oxidation rate had fallen by 51%. In contrast fat oxidation increased 4.9-fold during the same period. As a result, the RCOP occurred after 285 min of exercise (Figure 1B – 45 min).

**Figure 2:**
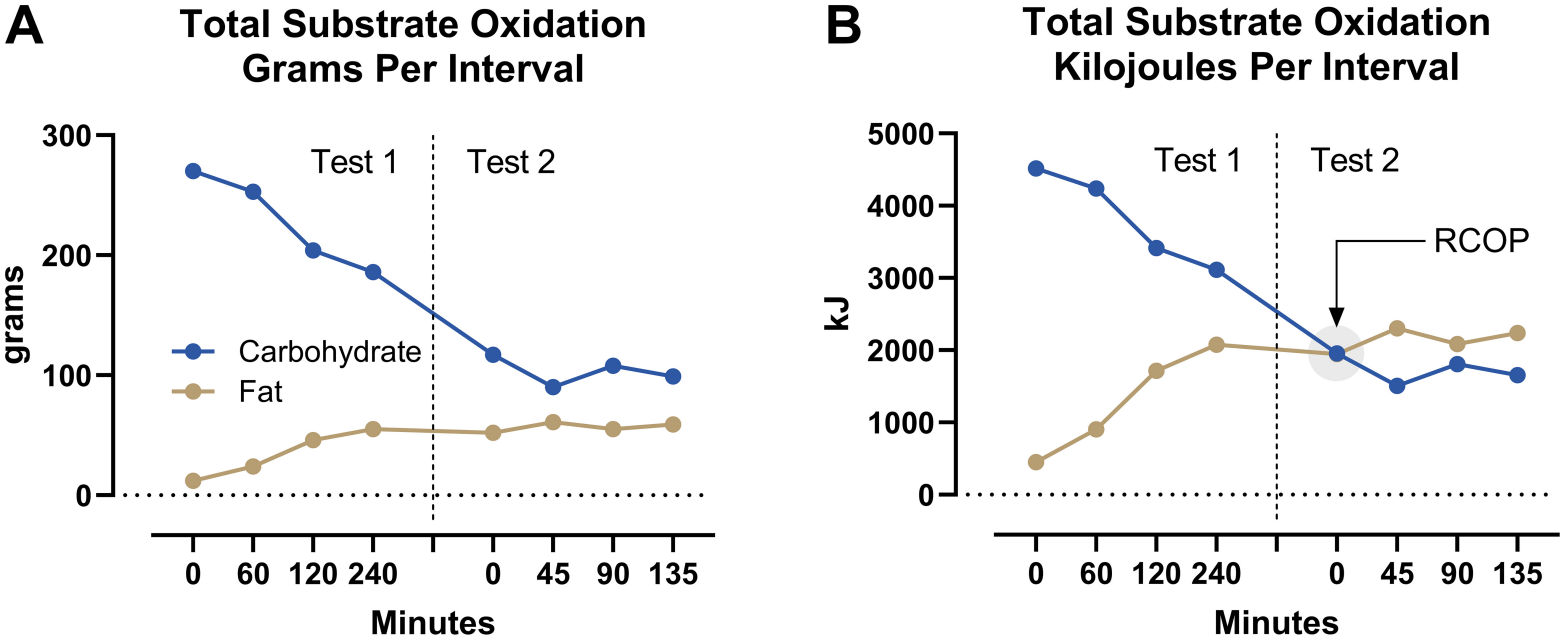
Total substrate oxidation. The carbohydrate and fat substrates were measured in g **(A)** and kJ **(B)** per measurement interval during the cycling (Test 1) and running (Test 2) legs of the simulated Ironman Triathlon.

Figure 2 depicts the cumulative amounts of CHO and fat oxidized for each measurement interval during the simulated Ironman Triathlon. Calculated total CHO oxidation during the 375 min of exercise was 1,327 g (22,207 kJ) whereas, total fat oxidation was 365 g (13,722 kJ). Despite a high rate (>80 g/h) of CHO ingestion during both the cycling and running legs, total CHO oxidation per interval fell progressively whereas, the contribution of fat to total energy expenditure increased progressively (Figure 2A). As a result, total substrate oxidation from fat expressed as kJ exceeded that from CHO after 45 min of the marathon run (Figure 2B).

## Discussion

Importantly, exercise performance measured as VO_2_ did not fall during the final 135 min of the marathon run even though total energy from fat oxidation (228 g; 8,572 kJ) was 24% greater than that from CHO oxidation (414 g; 6,927 kJ). As a result, ~55% of the total energy expenditure during the final 2.25 h of the simulation was provided by fat oxidation. This confirms a finding in a previous 6-h cycling simulation that “fat metabolism, even in conditions of very high glucose ingestion, as practiced in competitions, may cover a substantial part of the energy expenditure in certain phases of prolonged races” [Klaris et al. (18), p. 7].

However, this conflicts with the belief that world-class performances in athletic events competed at 60%−85% VO_2max_ can only be achieved by high rates of CHO oxidation once muscle glycogen concentrations are reduced (10, 19). Reviewing these data, Christensen (16) acknowledged: “Glycogen depletion and localization within the muscle fibers can effect muscle function (20). Nevertheless, of the three 10.5 km run loops on public road, the last one was the fastest which does not support any attainment of a ‘critical' low level of muscle glycogen affecting muscle function at the given exercise intensity” [Christensen (16), p. 6]. Rather it seems that moderately high rates of fat oxidation should be able to substitute for CHO oxidation even at higher exercise intensities (10).

Finally, the calculated amount (1,327 g) of CHO oxidized during this simulation exceeds by a factor of at least 50% the amount of CHO stored as glycogen at the start of exercise in elite male athletes. The maximum total muscle glycogen stores in elite athletes would be ~800 mmol/kg dry muscle (21) for a total store of about 620 g in an athlete who uses 17 kg of leg muscles (wet weight) when running. The liver provides an additional 75–90 g (22). The study of Coyle et al. (23) also suggests a maximum total muscle glycogen content of ~560 g and that of Gonzalez et al. (24), ~740 g. Others (25) have also identified this paradox that the estimated CHO requirement to complete the Ironman Triathlon “exceeds the whole-body carbohydrate content (24).” Thus, the question: from where does the additional 480 g of CHO arise?

The most obvious answer would be from ingested CHO since athletes are now encouraged to ingest at least 90–120 g CHO/hr during these competitions (26). Elsewhere, we (10) have shown that ingesting CHO at 144 g/h produces a total cumulative CHO oxidation of 137 g during the first 2 h (48% of ingested dose) followed by an hourly oxidation rate of 1.8 g/min (108 g/h) thereafter. Thus, during a 7-h Ironman Triathlon, an athlete ingesting CHO at a rate of 144 g/h would ingest a total of 1,008 g CHO and oxidize a maximum of 677 g CHO (68% of the ingested CHO).

Whilst this additional CHO intake would balance the CHO equation for this case study, it fails to explain two historical observations:

Even as recently as 2010, Hawaiian Ironman triathletes typically consumed carbohydrates at much lower rates (~60 g/h) (27) during the marathon leg compared to current practices (26). Despite this, the best marathon run times in the Hawaiian Ironman Triathlon (11, 28) in those years were comparable to those achieved by modern triathletes who now ingest carbohydrates at significantly higher rates. This raises a critical question: first, if carbohydrate availability is essential for sustaining high rates of carbohydrate oxidation after 4 or more hours of exercise (2, 29), how were the leading athletes in those earlier years able to sustain high rates of CHO oxidation during the run phase of the Ironman under conditions of much lower exogenous CHO intake? Second, if CHO is the preferred fuel for performance during prolonged exercise of intermediate intensity, typified by the Ironman Triathlon, why does the RCOP exist? According to current doctrine, the RCOP must be associated with impaired, not enhanced performance during the Ironman Triathlon.

## Conclusions

Our secondary analysis of substrate oxidation data published by Christensen (16) confirms the importance of high rates of fat oxidation to achieve world-record Ironman triathlon performance. Even in the presence of a high rate of CHO ingestion in the simulation, rates of fat oxidation increased progressively during exercise (Figures 1, 2), providing 55% of the total energy expenditure during the final 135 min of the simulation. The RCOP, defined here as the point at which fat oxidation exceeded CHO oxidation, was observed after 285 min of exercise. Notably, this occurred prior to the final 10.5-km lap of the run, during which the athlete recorded his fastest segment. While this temporal association suggests that high fat oxidation was not detrimental to performance, no causal inference can be drawn from this case study.

The study also shows that in the absence of high rates of exogenous CHO ingestion, it is difficult to explain total CHO oxidation during the Ironman Triathlon based on known total CHO stores present in liver and skeletal muscles at the start of exercise. However, as this is a single-subject case study, these findings should be interpreted as hypothesis-generating, warranting further research to determine the prevalence, physiological underpinnings, and performance implications of the RCOP across broader athlete populations.
